# Short‐ and long‐interval intracortical inhibition in EPM1 is related to genotype

**DOI:** 10.1111/epi.17466

**Published:** 2022-12-01

**Authors:** Katri Silvennoinen, Laura Säisänen, Jelena Hyppönen, Saara M. Rissanen, Pasi A. Karjalainen, Sasha D'Ambrosio, Diego Jimenez‐Jimenez, Sara Zagaglia, John C. Rothwell, Simona Balestrini, Sanjay M. Sisodiya, Petro Julkunen, Esa Mervaala, Reetta Kälviäinen

**Affiliations:** ^1^ Kuopio Epilepsy Center, Neurocenter Member of ERN EpiCARE, Kuopio University Hospital Kuopio Finland; ^2^ Department of Clinical and Experimental Epilepsy UCL Queen Square Institute of Neurology London UK; ^3^ Chalfont Centre for Epilepsy UK; ^4^ Department of Clinical Neurophysiology, Kuopio Epilepsy Center, Neurocenter Member of ERN EpiCARE, Kuopio University Hospital Kuopio Finland; ^5^ Department of Applied Physics University of Eastern Finland Kuopio Finland; ^6^ Dipartimento di Scienze Biomediche e Cliniche "L. Sacco" Università degli Studi di Milano Milan Italy; ^7^ Sobell Department of Motor Neuroscience and Movement Disorders UCL Queen Square Institute of Neurology London UK; ^8^ Neuroscience Department Member of ERN EpiCARE, Meyer Children Hospital Florence Italy; ^9^ Institute of Clinical Medicine, School of Medicine, Faculty of Health Sciences University of Eastern Finland Kuopio Finland

**Keywords:** LICI, progressive myoclonic epilepsy, SICI, transcranial magnetic stimulation, Unverricht‐Lundborg disease

## Abstract

**Objective:**

Progressive myoclonic epilepsy type 1 (EPM1) is caused by biallelic alterations in the *CSTB* gene, most commonly dodecamer repeat expansions. Although transcranial magnetic stimulation (TMS)–induced long‐interval intracortical inhibition (LICI) was previously reported to be normal in EPM1, short‐interval intracortical inhibition (SICI) was reduced. We explored the association between these measures and the clinical and genetic features in a separate group of patients with EPM1.

**Methods:**

TMS combined with electromyography was performed under neuronavigation. LICI was induced with an inter‐stimulus interval (ISI) of 100 ms, and SICI with ISIs of 2 and 3 ms, and their means (mSICIs) were expressed as the ratio of conditioned to unconditioned stimuli. LICI and mSICI were compared between patients and controls. Nonparametric correlation was used to study the association between inhibition and parameters of clinical severity, including the Unified Myoclonus Rating Scale (UMRS); among patients with EPM1 due to biallelic expansion repeats, also the association with the number of repeats was assessed.

**Results:**

The study protocol was completed in 19 patients (15 with biallelic expansion repeats and 4 compound heterozygotes), and 7 healthy, age‐ and sex‐matched control participants. Compared to controls, patients demonstrated significantly less SICI (median mSICI ratio 1.18 vs 0.38; *p* < .001). Neither LICI nor SICI was associated with parameters of clinical severity. In participants with biallelic repeat expansions, the number of repeats in the more affected allele (greater repeat number [GRN]) correlated with LICI (rho = 0.872; *p* < .001) and SICI (rho = 0.689; *p* = .006).

**Significance:**

Our results strengthen the finding of deranged γ‐aminobutyric acid (GABA)ergic inhibition in EPM1. LICI and SICI may have use as markers of GABAergic impairment in future trials of disease‐modifying treatment in this condition. Whether a higher number of expansion repeats leads to greater GABAergic impairment warrants further study.

**Key points**

- We studied γ‐aminobutyric acid (GABA) ergic inhibition in patients with progressive myoclonic epilepsy type 1 (EPM1) using paired‐pulse transcranial magnetic stimulation–electromyography (TMS‐EMG), confirming the impairment of short‐interval intracortical inhibition (SICI); also long‐interval intracortical inhibition (LICI) was impaired in some individuals.
- In patients with EPM1 due to biallelic repeat expansion variants, the number of repeats in the longer expansion correlated with the degree of impairment in LICI and SICI.
- SICI and/or LICI may have use as markers of GABAergic impairment in possible future trials of disease‐modifying treatment in EPM1.

## 1 INTRODUCTION

Progressive myoclonic epilepsy type 1 (EPM1, OMIM 254800), also known as Unverricht‐Lundborg disease, typically presents between the ages of 6 and 16 years and is characterized by myoclonus, which is stimulus sensitive and action activated, and generalized tonic‐clonic seizures.^1^ These symptoms may be treated with antiseizure medications (ASMs).^1^ Unfortunately, no disease‐modifying treatments are available at present and the disease course is progressive, with worsening myoclonus and ataxia.^1^ Most patients ultimately lose the ability to mobilize without aids and have difficulties with many activities of daily living (ADLs).^2^ However, there is significant individual variability in the disease course.^1^

EPM1 is an autosomal recessive condition caused by biallelic pathogenic variants in the *CSTB* gene, which encodes cystatin B, a protease inhibitor.^3^, ^4^ Approximately 90% of *CSTB* alleles associated with EPM1 involve an unstable dodecamer repeat expansion in the promoter region of the gene (reported range 30–125 repeats, in contrast with 2–3 in healthy individuals).^3^ The majority of patients have biallelic repeat expansion variants, whereas a minority are compound heterozygotes, with one copy of a repeat expansion variant and one point mutation in the *CSTB* gene.^3^ Pathogenic repeat expansions lead to reduced expression of *CSTB*; the point mutations associated with disease also cause loss of function.^3^ Exactly how *CSTB* loss of function causes the disease remains incompletely understood. Possible mechanisms include neurodegeneration mediated via loss of neuroprotective effects of cystatin B, and disinhibition of cathepsins.^3^ Recently, Di Matteo et al. demonstrated that CSTB acts as a signaling molecule that appears to be integral for the migration of inhibitory interneurons to the cortex.^5^ This could provide a mechanism for the findings of Buzzi et al., who showed, in a knock‐out mouse model, that reduced thickness of sensorimotor cortex was associated with reduced vesicular γ‐aminobutyric acid (GABA) transporter (VGAT) signal.^6^ Paired‐pulse inhibition was reduced, suggesting impaired GABA_A_ergic and GABA_B_ergic inhibition.^6^ In a single human postmortem sample, the motor cortex showed thinning and reduced VGAT‐labeled GABA terminals.^6^ These studies suggest reduced GABAergic signaling as one possible disease mechanism in EPM1.

For successful development of pathology‐reversing treatments, it is essential to understand the underpinnings of interindividual phenotypic variability. Quantifiable markers of such variability might be employed in stratifying patients in treatment trials and potentially even as outcome measures.

Transcranial magnetic stimulation (TMS) is a non‐invasive means of studying cortical excitability and inhibition of (mainly GABAergic) neurotransmission,^7^ and, therefore, has the potential for biomarker use in conditions such as EPM1, where these processes may be impaired. Our group has shown previously that, compared to controls, patients with EPM1 due to biallelic pathogenic repeat expansion have a prolonged cortical silent period (CSP), implying enhanced GABA_B_ergic inhibition^8^; the degree of CSP prolongation correlated with the size of the repeat expansion in the more affected allele (henceforth referred to as greater repeat number [GRN]), and independently predicted the severity of myoclonus.^2^ Furthermore, in a sample of compound heterozygous individuals with EPM1, whose clinical phenotype is also more severe, the prolongation of CSP appeared to be accentuated.^9^

Subsequently, paired‐pulse TMS was performed in a group of 10 patients with EPM1 from Italy, in whom no difference in long‐interval intracortical inhibition (LICI) was shown compared to healthy controls.^10^ In contrast, short‐interval intracortical inhibition (SICI) was reduced,^10^ implying impairment in GABA_A_ergic inhibition.^6^, ^11^

We performed paired‐pulse TMS with the aim of exploring the possible associations between these TMS measures of GABAergic inhibition and clinical and genetic features. We hypothesized that SICI would be impaired also in our patient group, and that this as well as possible impairment in LICI might be associated with a more severe phenotype or genotype. Specifically, we expected that compared to patients with biallelic expansion repeat, compound heterozygous patients would show more inhibition impairment. Among those with biallelic expansion repeats, we hypothesized that the degree of impairment would be associated with the number of repeats in the more affected allele (GRN).

## 2 METHODS

### 2.1 Participants

The subject group consisted of patients with genetically confirmed EPM1 and visiting our center to participate in a study of ambulatory monitoring of myoclonus using surface electromyography (EMG) and three‐dimensional accelerometry.^12^ Inclusion criteria were the ability to provide written informed consent to the procedure and a magnetic resonance imaging (MRI) scan within the preceding 5 years or an ability to undergo an MRI prior to the experiment. Exclusion criteria included pregnancy or the presence of any intracranial metallic devices or implants.

The genetic diagnoses had been confirmed at the University of Helsinki Molecular Laboratory, either on a research^2^ or clinical basis, and this and other clinical information was collected systematically as a part of this study.

All patients underwent assessment with the Unified Myoclonus Ranking Scale (UMRS)^13^ at the same study visit, assessed by an experienced researcher (JH). Clinical data were obtained by means of interview and from medical records. Hand dominance was determined using the Waterloo Handedness Questionnaire.^14^

Written informed consent was obtained from all participants. This study was approved (statement 410/2019) by the ethics committee of the North Savo Hospital District.

In view of the rarity of the condition, one patient from the UK with EPM1 was recruited through the outpatient epilepsy clinic to take part in a study of epilepsy genetics (Camden & Kings Cross Research Ethics Committee reference 11/LO/2016). Genetic and clinical data, except for UMRS, were collected as for the other patients.

In total, 21 patients (12 female) took part (Table 1). Seven healthy individuals (four female) were recruited as control participants (London‐South East Research Ethics Committee reference 15/LO/1642; Cortical Excitability in Neurological Genetic Conditions), chosen to match the patients with respect to age and sex distributions (considered separately). Exclusion criteria included pregnancy and the presence of any intracranial metallic devices or implants.

**TABLE 1:** Characteristics of patients with EPM1 (*n* = 21)

Age at onset in years, median (range)	9 (6–11)
Duration of disease in years, median (range)	19 (8–42)
Severity of disease/ambulation status, *n* (%)	
Completely ambulatory	10 (47.6)
Occasional wheelchair use	3 (14.3)
Completely wheelchair‐dependent	8 (38.1)
Genetic status	
Biallelic expansion, *n* patients (%)	16 (76.2)
Greater repeat number, median (range)	72.5 (61–85)
Compound heterozygote^a^, *n* patients (%)	5 (23.8)
c.202C > T (p.R68X)	4 (19.0)
c.218_219delTC (p.L73fsX3)	1 (4.8)
Greater repeat number, median (range)	70 (70–100)
Number of current ASMs, median (range)	3 (2–6)
Individual ASMs, *n* (%) patients	
Valproate	21 (100)
Levetiracetam	12 (57.1)
Brivaracetam	8 (38.1)
Clonazepam	8 (38.1)
Clobazam	6 (28.6)
Perampanel	4 (19.0)
Topiramate	4 (19.0)
Piracetam	3 (14.3)
Zonisamide	2 (9.5)
UMRS Functional Test score^b^ median (range)	8.5 (1–28)
UMRS Stimulus Sensitivity score^b^ median (range)	3 (0–12)
UMRS Action myoclonus score^b^ median (range)	45 (3–123)
UMRS Negative myoclonus severity score^a^ median (range)	0 (0–2)

^a^ All with a single point mutation and one repeat expansion.

^b^ Available for 20 patients. Abbreviations: ASM, antiseizure medication; EPM1, progressive myoclonic epilepsy type 1; UMRS, Unified Myoclonus Ranking Scale.

### 2.2 TMS equipment

Neuronavigated TMS (eXimia 3.2; Nexstim Plc., Helsinki, Finland) was performed at the Department of Clinical Neurophysiology, Kuopio University Hospital (Kuopio, Finland). Individual structural MRI studies were used for neuronavigation.

For one patient and all control participants, measurements were performed at the Chalfont Centre for Epilepsy, Buckinghamshire, UK, under University College London Hospitals (UCLH) clinical governance. Neuronavigation was performed using Brainsight software (Rogue Research, Montreal, Canada). For the person with EPM1 and four controls, individual MRI studies were used. For the remaining three controls, a template MRI provided by the software was used.

Both neuronavigation systems involved infrared sensor units, attached to the subject by headband (eXimia) or glasses (Brainsight), for co‐registering the TMS coil and with the participant's head within the reference space of the individual structural MRI or the template.

At both centers, TMS was performed using two Magstim 200 stimulators connected via a BiStim unit generating a monophasic TMS‐pulse. Pulses were delivered through a 70 mm figure‐of‐eight coil (Alpha coil, Magstim, Whitfield, UK).

### 2.3 TMS procedure

Participants sat comfortably in a reclining chair with a head rest. Surface EMG was measured from the dominant first dorsal interosseus (FDI) muscle using a belly‐tendon montage.

Using MRI guidance, the search for the hotspot was started in the omega area of the precentral gyrus. Once an intensity sufficient to provoke motor evoked potentials (MEPs) was identified, the exact stimulation location producing the largest MEPs was identified. Following this, the coil orientation was altered by 45 degrees in each direction, with several stimuli delivered at each position. Following identification of the hotspot, the resting motor threshold (rMT) was identified as the minimum intensity able to produce MEPs in 5 of 10 trials and was quantified as percentage of the maximum stimulator output (%‐MSO).^7^

For two patients, rMT was >100%‐MSO and they were excluded from further testing. For the rest (*n* = 19), the median rMT was 66%‐MSO, which was significantly higher than that of controls: 51% (Mann‐Whitney *U*, *p* = .004) (Table 2).

**TABLE 2:** Single‐ and paired‐pulse parameters in patients and controls

	EPM1 (*n* = 19)	Controls (*n* = 7)	*p*‐value
rMT (%‐MSO)	66 (48–85)	51 (37–63)	.004
TS amplitude (mV)	1.33 (0.18–2.84)	1.00 (0.74–4.73)	1.000
LICI ratio	0.04 (0.00–2.49)^a^	0.04 (0.04–0.24)	.701
SICI 2 ms ratio	1.16 (0.64–2.34)^a^	0.42 (0.12–0.89)	<.001
SICI 3 ms ratio	1.14 (0.67–1.69)^b^	0.22 (0.05–0.64)	<.001
mSICI	1.18 (0.68–2.01)^a^	0.38 (0.11–0.77)	<.001
cICI	0.62 (0.35–1.96)^b^	0.29 (0.08–0.40)	<.001

*Note*: Data are median and range.

Abbreviations: LICI, long‐interval intracortical inhibition; MSO, maximum stimulator output; mV, millivolt; rMT, resting motor threshold; SICI, short‐interval intracortical inhibition; TS, test stimulus. mSICI mean short‐interval intracortical inhibition. cICI refers to the average of (combined) mSICI and LICI ratios. *p*‐value refers to Mann‐Whitney *U* test.

^a^ *n* = 18.

^b^ *n* = 17.

For the paired‐pulse protocols, the intensity of the unconditioned stimulus was set at 120% rMT. To uniformly achieve unconditioned MEPs of around 1 mV, several trial MEPs were produced at 120% rMT. If this resulted in significantly larger MEPs, the intensity of the unconditioned stimulus was lowered slightly. No statistically significant difference between the groups was observed in the amplitude of the test stimulus (Mann‐Whitney *U*, *p* > .05).

For SICI, the intensity of the conditioning stimulus (CS) was set at 70%. Although SICI was described originally to be maximal at CS 80% rMT,^15^ CS 70% rMT consistently elicits SICI^15^, ^16^, ^17^; we chose this intensity to avoid contamination by short‐interval intracortical facilitation.^18^ For SICI, inter‐stimulus intervals (ISIs) of 2 and 3 ms were used. For LICI, the CS was 120% rMT and the ISI was 100 ms (Figure S1).

The stimuli were delivered in blocks. The order of the blocks was randomized for each participant prior to starting. The paired‐pulse blocks consisted of 30 stimuli each. The unconditioned stimuli were split into a total of three to four blocks (10 stimuli each).

An experienced researcher constantly monitored the position of the coil on a second screen, as well as the EMG trace for muscle contraction. Patients were monitored for the presence of myoclonus (sustained myoclonus would have led to discontinuation of the experiment). All participants were monitored for any signs of discomfort. If a participant began to appear drowsy, the stimulation was briefly interrupted, and the participant was engaged in a brief conversation to raise their level of alertness.

### 2.4 Data processing

MEPs were visualized and measured using eXimia 3.2 (Nexstim Plc, Helsinki, Finland) or Signal (Cambridge Electronics Design, Cambridge, UK) software. All trials were checked visually to exclude those with baseline muscle activity.

### 2.5 Statistical analyses

LICI and SICI were expressed as the ratio of median conditioned MEP amplitude to the median unconditioned MEP amplitude. SICI was originally described using ISIs of both 2 and 3 ms^15^; both are commonly reported in studies using TMS in epilepsy‐associated conditions.^19^ It is plausible that there is some inter‐individual variability as to which of these ISIs is optimal. Accounting for this, as well as to obtain a single value for SICI, we averaged the ratios for both ISIs to yield the mean SICI (mSICI), an approach described previously.^20^ One patient wanted to stop testing before completion of 3 ms SICI, so for this individual, 2 ms SICI was used in place of the mSICI. We also report data for both ISIs separately.

For a general assessment of intracortical inhibition, LICI and mSICI were further averaged to yield a novel measure, combined intracortical inhibition (cICI). A receiver‐operator characteristic (ROC) analysis was performed to study the ability of this parameter to differentiate between patients and controls.

Due to a generally non‐normal distribution, the central tendency was expressed as median, and between‐group comparisons with respect to continuous variables were done with non‐parametric Mann‐Whitney *U* test.

In patients, correlation was tested between LICI and SICI, and age, duration of EPM1, UMRS subscores, number of ASMs used, use of benzodiazepines or topiramate, ambulation status, rMT, and GRN (in those with biallelic expansion repeats). Correlation tests were chosen as appropriate based on the data distribution. In the case of normal distribution, Pearson's correlation coefficient was used; for non‐normal distribution, Spearman's rho was used. A two‐tailed value of *p* ≤ .05 was considered statistically significant.

Statistical analyses were performed using SPSS version 27 (IBM Corp, Armonk, NY, USA). Prism 9 (GraphPad Software, San Diego, CA, USA) was used for the graphic presentation of the data.

## 3 RESULTS

The median age was 26 years (range 19–21) in patients and 33 (range 31–54) in controls. The difference in distributions was not statistically significant (*p* = .249). Three patients (14%) and one control (14%) were left‐handed.

Among patients, 16 had biallelic expansion variants, whereas 5 were compound heterozygotes, with both an expansion variant and a point mutation (Table 1). Both point mutations identified are considered pathogenic due to predicted protein truncation and functional evidence for reduced *CSTB* expression and are associated with EPM1.^3^

Fifteen patients (71.4%) took ASMs with possible SICI‐promoting effects (clonazepam and/or clobazam in 14 patients and topiramate in 1 patient).^22^

**FIGURE 1:**
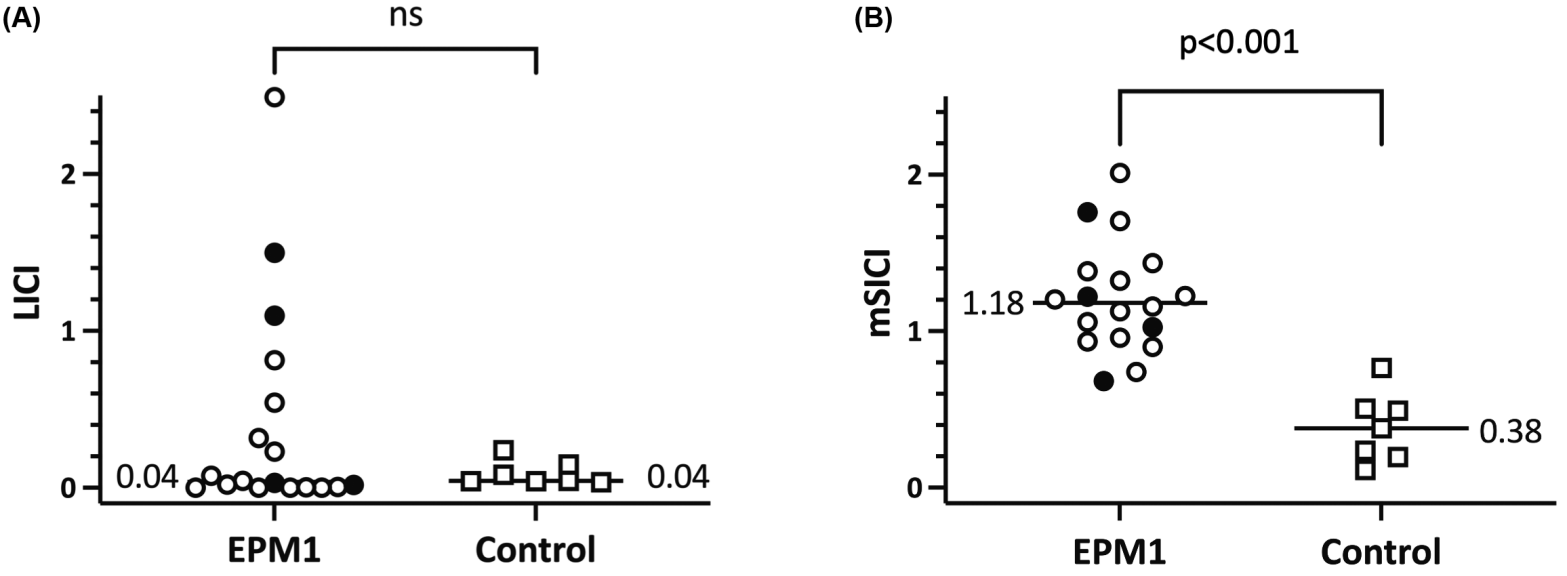
Comparison of long‐interval intracortical inhibition (LICI) (A) and mean short‐interval intracortical inhibition (mSICI) (B) between patients with progressive myoclonic epilepsy type 1 (EPM1) and controls. LICI and SICI are expressed as the amplitude ratio of conditioned to unconditioned stimuli; for SICI, this is averaged over the two inter‐stimulus intervals (ISIs). All individual data points are displayed; for EPM1, compound heterozygotes are indicated in black. Horizontal lines present median for each group. Pairwise comparisons refer to Mann‐Whitney *U* test. N's, not significant.

There was no statistically significant difference in LICI between patients and controls (Table 2). As assessed by an LICI ratio <1.0, LICI was present in all controls and in 15 of 18 patients. In contrast, compared to controls, patients showed significantly reduced SICI at both 2 ms and 3 ms ISIs, as well as the average of these two (mSICI; Table 2, Figure 1). Considering both patients and controls, or just patients, no differences in LICI or SICI were seen in the left‐handed compared to the right‐handed (Figure S2).

**FIGURE 2:**
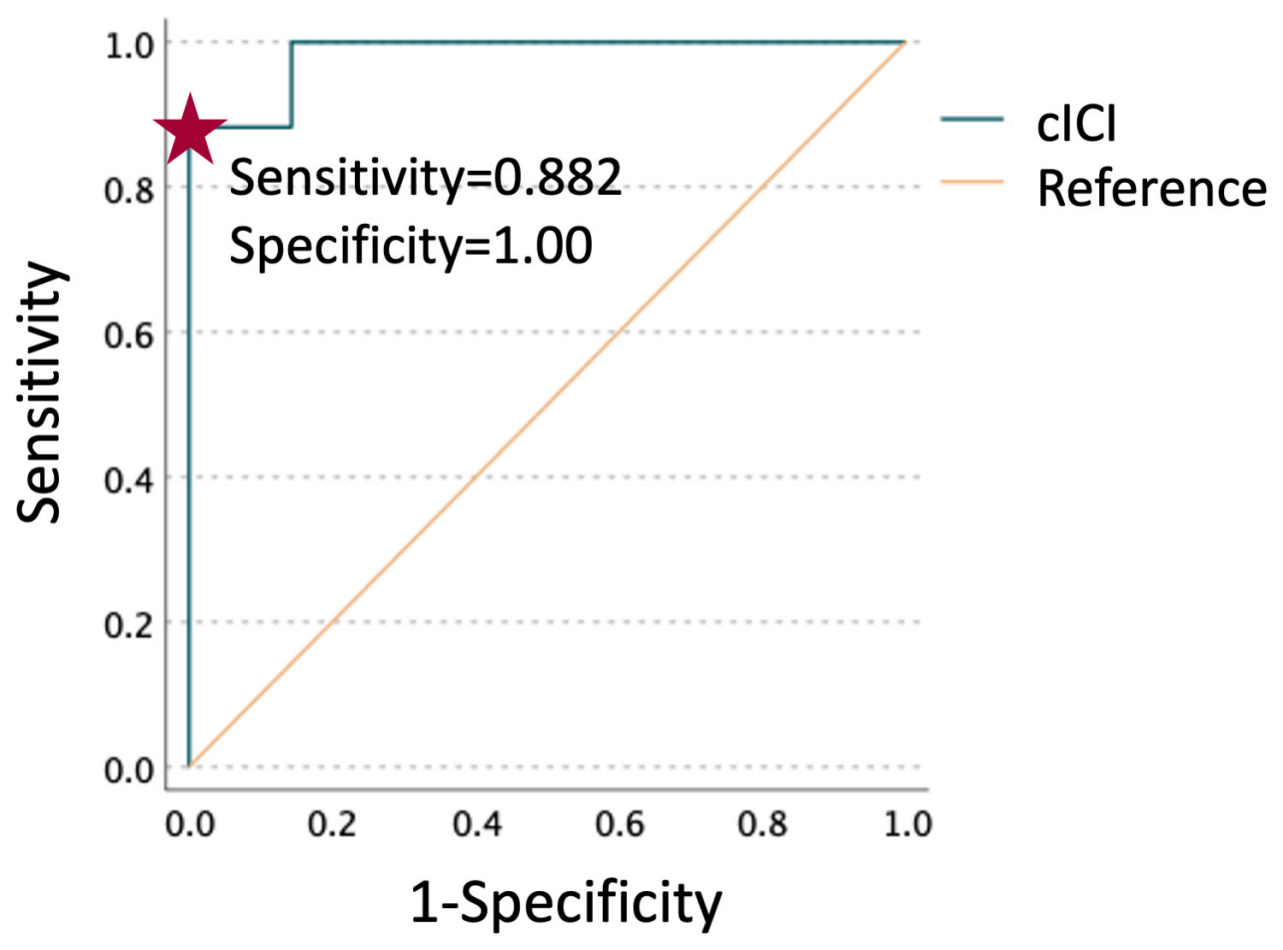
Receiver‐operator characteristic (ROC) curve for differentiating between patients and controls based on combined short‐ and long‐interval intracortical inhibition (cICI). cICI is expressed as the average of the ratios of conditioned to unconditioned stimuli amplitudes. Star denotes selected cutoff of 0.425, yielding a sensitivity of 0.882 and specificity of 1.00. Area under the curve (AUC) = 0.983.

Both LICI and SICI showed inter‐individual differences. There was a significant correlation between LICI and SICI (rho = 0.689, *p* = .002). To combine these in a single measure of inhibition, for each patient, LICI and SICI were also averaged to yield combined inhibition (or cICI). Compared to controls, cICI was significantly reduced in patients (Table 2). ROC analysis (Figure 2) showed that cICI could reliably distinguish between patients and controls. The optimal cutoff was identified as 0.425, yielding a sensitivity of 0.882, specificity of 1.000, and area under the curve (AUC) = 0.983.

Among all patients there was a significant negative correlation between age and LICI ratio, implying a greater impairment of inhibition among younger patients (Table 3; Figure S3, rho = −0.500; *p* = .035); this did not persist when considering only those with biallelic repeat expansions (Table S1). No significant correlation was observed between age and either mSICI (Table 3) or cICI (rho = −0.373, *p* = .140). There was no significant correlation between either LICI or SICI, and disease duration, number of ASMs, ambulation status, any of the UMRS subscores, or rMT (Table 3). Furthermore, there was no significant difference in either LICI or SICI by use of regular benzodiazepines or topiramate (LICI: *p* = .775; SICI: *p* = .831).

**TABLE 3:** Spearman correlation between LICI and SICI and clinical parameters and rMT in all patients with EPM1

Parameter	LICI (*n* = 18)	mSICI (*n* = 18)
Age	rho = −0.500; *p* = .035*	rho = −0.301; *p* = .224
Disease duration	rho = −0.387; *p* = .112	rho = −0.267; *p* = .284
Number of ASMs	rho = 0.062; *p* = .808	rho = 0.011; *p* = .965
Ambulation status	rho = −0.016; *p* = .949	rho = 0.230; *p* = .358
UMRS Functional Test score^a^	rho = 0.384; *p* = .128	rho = 0.162; *p* = .533
UMRS Stimulus Sensitivity score^a^	rho = 0.001; *p* = .996	rho = −0.074; *p* = .777
UMRS Action myoclonus score^a^	rho = 0.025; *p* = .926	rho = 0.000; *p* = 1.000
UMRS Negative myoclonus severity score^a^	rho = −0.096; *p* = .715	rho = −0.113; *p* = .666
rMT	rho = 0.076; *p* = .766	rho = 0.201; *p* = .424

Abbreviations: ASM, antiseizure medication; EMP1, progressive myoclonic epilepsy type 1; LICI, long‐interval intracortical inhibition; mSICI mean short‐interval intracortical inhibition; rMT, resting motor threshold; UMRS, Unified Myoclonus Ranking Scale.

* Statistically significant (*p* ≤ .05).

^a^ *N* = 17.

At group level, there was no difference between compound heterozygotes and those with biallelic repeat expansions for either LICI (Mann‐Whitney *U*, *p* = .277), or SICI (Mann‐Whitney *U*, *p* = .798; Figure 1).

**FIGURE 3:**
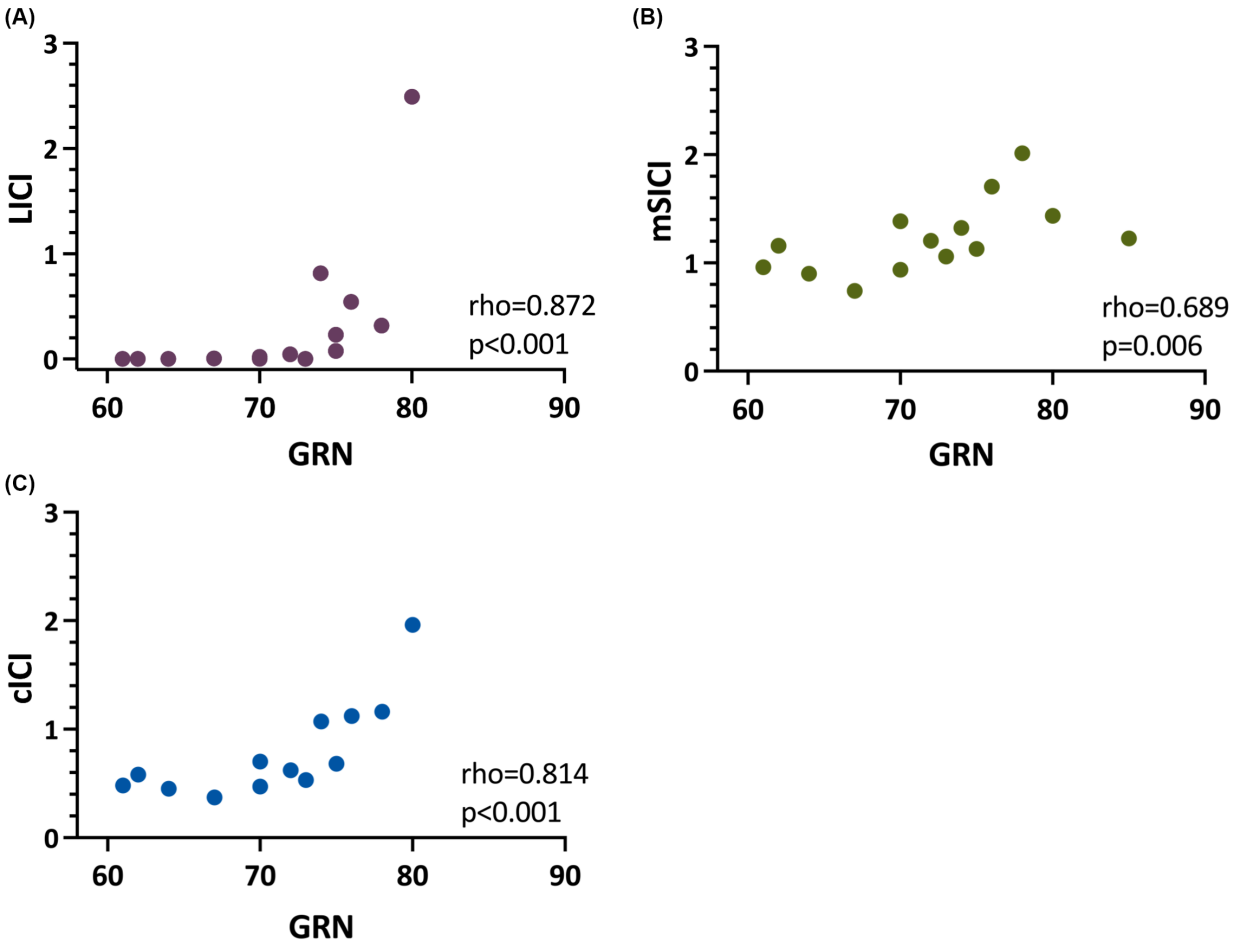
Correlation between greater repeat number (GRN) and long‐interval intracortical inhibition (LICI) (A), mean short‐interval intracortical inhibition (mSICI) (B), and combined intracortical inhibition (cICI) (C). Data are shown for individuals with biallelic repeat expansions.

Among those with biallelic repeat expansions, GRN correlated significantly with both the degree of LICI (Figure 3A; rho = 0.872, *p* < .001), mSICI (Figure 3B; rho = 0.689, *p* = .006), and cICI (Figure 3C; rho = 0.814, *p* < .001).

## 4 DISCUSSION

In this paired‐pulse TMS study in EPM1, a combination of LICI and SICI (cICI) robustly differentiated patients from controls. Both LICI and SICI showed inter‐individual variability. We found no association between LICI or SICI and clinical severity. The number of compound heterozygous patients was small. However, among patients with EPM1 due to biallelic repeat expansions, a greater number of repeats in the longer expansion was associated with greater SICI and LICI ratios, that is, weakened inhibition.

Advances in the use of TMS as a biomarker in the genetic epilepsies are hindered by a lack of replication studies and small participant number due to rarity of the conditions.^20^ We confirm the previous findings of Canafoglia et al. of impaired SICI.^10^ Canafoglia et al. reported LICI to be preserved.^10^ Although on the group level, we showed no statistically significant difference between patients and controls, in some patients, LICI was clearly impaired.

Our group previously showed prolongation of CSP in patients with EPM1, suggesting *enhanced* GABA_B_ergic cortico‐spinal signaling.^8^ On the other hand, a TMS/EEG (electroencephalography) study showed reduced amplitudes of N100 and P180, suggesting *decreased* GABA_B_ inhibition, at least cortico‐cortically.^23^ Overall, our current findings are congruent with the preclinical evidence of Buzzi et al. of impaired cortical GABA_A_ergic and GABA_B_ergic inhibition in EPM1.^6^ Enhancing GABA_A_ergic inhibition by agents such as the benzodiazepine clonazepam has an established role in the symptomatic treatment of myoclonus in EPM1.^1^ Reversal of the impairment in GABAergic inhibition might serve as a target for the development of disease‐modifying treatment in EPM1, in which case SICI and/or LICI might be potential markers of treatment effect.

Although EPM1 was among the first epilepsies with a determined genetic cause, identified in 1996,^4^ understanding of the basis of inter‐individual variability remains limited.^1^ Observational evidence suggests that compared to those with biallelic repeat expansions, the minority of patients who have EPM1 due to compound heterozygous mutations may have a more severe phenotype, as evidenced by earlier age at onset and the severity of myoclonus.^1^ However, in a study by Canafoglia et al, this could not be linked with a lower expression of *CSTB*.^24^ Despite the more severe phenotype in the eight compound heterozygotes included (each with one point mutation and one repeat expansion), there was no difference in *CSTB* expression compared to the 40 patients with biallelic repeat expansions; both groups showed reduced expression compared to controls.^24^

Among patients with biallelic repeat expansions, the association between phenotype severity and the size of the repeat expansion in EPM1 has been debated.^1^ In an early series of 28 patients, no association between age at onset and size of the longest, shortest, or average repeat was not shown.^25^ In a subsequent study of 66 patients from our group, Hyppönen et al. identified an association between the number of expansions in longer repeat (GRN) and action myoclonus severity measured by UMRS; there was also trend for earlier age at onset.^2^ These findings serve as a motivation to identify other measures that might aid in the understanding of the relationship between genotype and phenotype in EPM1.

Contrary to our hypothesis, we did not observe any significant association between either SICI or LICI, and the clinical severity parameters measured at a single time point, such as ambulation status or myoclonic severity as measured by UMRS. Regarding our second hypothesis, only four compound heterozygous patients completed the study, and in this small patient group, no difference in LICI or SICI could be established in comparison to those with biallelic repeat expansions. In contrast, in keeping with our third hypothesis, among patients with EPM1 due to biallelic repeat expansions, we identified a significant association between GRN and SICI and LICI.

It is compelling to consider that the association between a greater number of repeats and weaker inhibition as measured by SICI and LICI might be due to more repeats causing a more severe disruption of GABAergic signaling. This warrants further research.

Considering all patients, age was correlated significantly with LICI; also SICI showed some trend for less impairment with greater age. With our cross‐sectional study, variability in patient age is a possible confounding factor, as among patients with the worst clinical trajectory, those in later adulthood might be too unwell to take part in research. To further assess correlation with phenotype and age, it would be valuable to obtain longitudinal measurements to assess for possible changes over the course of disease progression. In any case, our results suggest that GABAergic impairment occurs relatively early in the disease.

Controlling for the possible effects of ASMs is a challenge in studies involving people with active epilepsy. With the caveat of the majority of previous drug effect data being derived from single‐dose studies in healthy controls, based on the existing literature, the ASMs taken by our patients would not be expected to impair LICI or SICI, or to enhance LICI at ISI 100 ms^25^, ^26^, ^27^, ^28^ (see Appendix S1 for a more detailed review). Diazepam, a GABA_A_ receptor allosteric modulator of the benzodiazepine family, paradigmatically enhances SICI^25^; it is unclear whether this effect is true for all benzodiazepines.^27^, ^29^ Topiramate has also been reported to enhance SICI.^21^ In our sample, no difference in SICI was observed associated with the use of benzodiazepines vs topiramate. In summary, we cannot exclude that differences in ASM therapy could have contributed to inter‐individual differences in LICI and SICI in our study. However, given the uniformity of ASM regimens, it seems unlikely that this would be a driving factor.^1^

In a previous study in healthy individuals, left‐handed individuals showed less SICI compared to the right‐handed^30^; another study failed to show any difference.^31^ Our sample included four left‐handed individuals (three patients and one control). Although we did not observe any such signal in our participants, we cannot exclude some effect of handedness in the inter‐individual variability in SICI or other TMS parameters, and we acknowledge this as a limitation.

EPM1 is a rare condition and the actual number of participants in our study remains small. In addition to increasing the number of patients, in the future it would be important to assess test–retest reliability. Although we do not think that this would have influenced the main findings of this study, the number of healthy controls could also have been greater. The strengths of our study include detailed genetic, clinical, and neurophysiological data on the patients and the use of neuronavigation to reduce intertrial variability.

In conclusion, a combination of LICI and SICI (cICI) robustly differentiated patients from controls. Both measures showed inter‐individual variability, and as such might be employed to quantify GABAergic impairment and measure its reversal in the context of possible future disease‐modifying treatments. However, such applications might be hindered by elevated rMT and difficulty in complying with the requirements of the experiment due to disability. In patients with biallelic repeat expansion variants, GRN correlated with the degree of impairment in LICI and SICI, suggesting that GABAergic impairment in EPM1 may be influenced by genetic severity. This warrants further research into the mechanisms by which GABAergic impairment arises as a result of loss of function of *CSTB*.

## AUTHOR CONTRIBUTIONS

KS: study design, data collection, analysis, drafting of the manuscript; LS: study design, data collection, interpretation of results; JH, SMR: study design, data collection; SD, DJJ, SZ: data collection; JCR, SB, SMS: supervision; PJ, EM, RK: study design, supervision. All authors: critical review and approval of the manuscript.

## CONFLICT OF INTEREST

RK has received speaker's honoraria from Eisai, Omamedical, Orion, Sandoz, Sanofi, and UCB; and honoraria for membership of the advisory boards/consultation of Eisai, Marinus Pharmaceuticals, Orion, and UCB. LS has received travel bursaries and PJ has received consulting fees and shares a patent with Nexstim Plc, the manufacturer of nTMS systems. UCB provided financial support for SD and SZ. UCB had no editorial control and no input or decision over the selection of authors or topics discussed. The remaining authors have no conflicts of interests.

## Supporting information


Appendix S1.
