# Supplementary material for: Short‐ and long‐interval intracortical inhibition in EPM1 is related to genotype
Source: Epilepsia. 2022 Dec 1;64(1):208–17. doi: 10.1111/epi.17466 (PMC10107775; doi:10.1111/epi.17466)
Supplement: Supplementary file 1 — Appendix S1. [file EPI-64-208-s001.pdf]

Supplementary material to

Short- and long-interval intracortical inhibition in EPM1 is related to genotype

Silvennoinen K, Säisänen L, Hyppönen J, Rissanen SM, Karjalainen PA, D'Ambrosio S, Jimenez-Jimenez D, Zagaglia S, Rothwell JC, Balestrini S, Sisodiya SM, Julkunen P, Mervaala E, Kälviäinen R

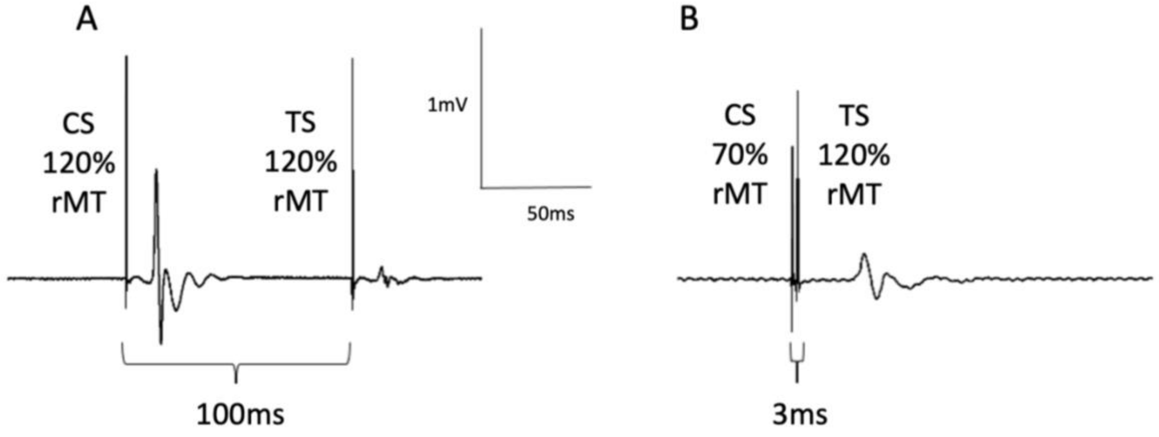

Supplementary Figure 1. Motor evoked potential (MEP) traces illustrating LICI (A) and SICI (B). In LICI (A), a conditioning stimulus (CS) evokes an MEP, but the MEP of a stimulus of a test stimulus (TS) of the same intensity applied 100ms later shows inhibition. In SICI (B), the inhibition is evoked by a subthreshold CS applied 2-3ms prior to the TS.

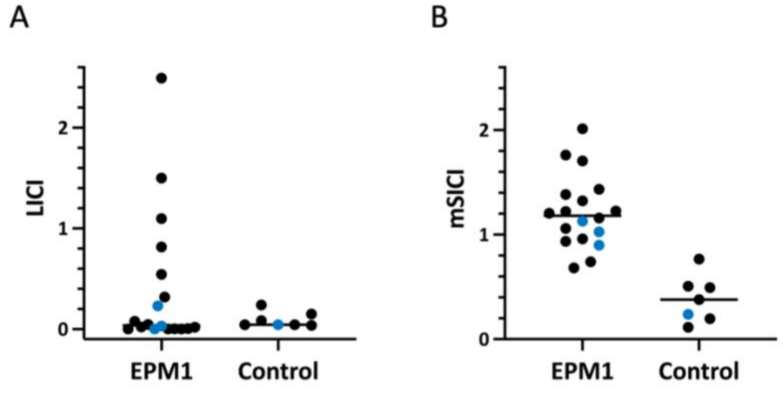

Supplementary Figure 2. LICI and SICI in patients and controls with left-handed individuals indicated in blue. LICI and SICI are expressed as the amplitude ratio of conditioned to unconditioned stimuli; for SICI this is averaged over the two ISIs. All individual data points are displayed. Horizontal lines present median for each group. No difference by handedness was seen in patients for either LICI or SICI.

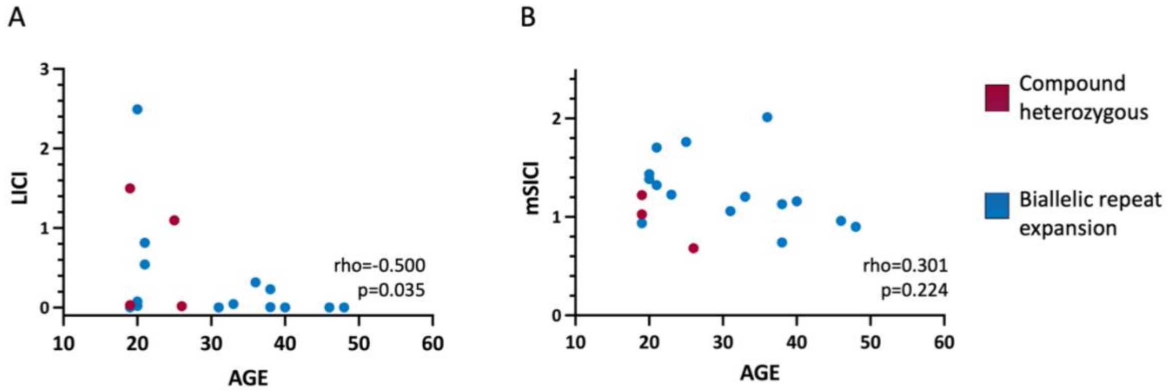

Supplementary Figure 3. Correlation between age and LICI (A) and mSICI (B). For all patients, LICI showed a significant correlation with age, whereas SICI did not (Table 3 main text). Considering only patients with biallelic repeat expansions (blue), correlation between age and neither LICI or SICI was significant (Supplementary Table 1).

Supplementary Table 1. Spearman correlation between LICI and SICI, and clinical parameters and rMT in patients with EPM1 due to biallelic repeat expansions

**Parameter**	**LICI (n=14)**	**mSICI (n=14)**
Age	rho= -0.482; p=0.081	rho= -0.472; p=0.088
Disease duration	rho=-0.423; p=0.132	rho=-0.469; p=0.091
Number of ASMs	rho=0.152; p=0.605	rho=0.182; p=0.532
Ambulation status	rho=-0.053; p=0.858	rho=-0.005; p=0.987
UMRS Functional Test score	rho=0.210; p=0. 472	rho=-0.064; p=0.828
UMRS Stimulus Sensitivity score	rho= -0.258; p=0.0.374	rho=-0.311; p=0.279
UMRS Action myoclonus score	rho=-0.147; p=0.615	rho=-0.275; p=0.342
UMRS Negative myoclonus severity score	rho= -0.376; p=0.186	rho=-0.351; p=0.219
rMT	rho= 0.162; p= 0.581	rho=0.194; p=0.505

Effects of antiseizure mediations used by the patients in this study on TMS parameters

In healthy subjects, levetiracetam and brivaracetam have no reported effects on SICI.[1–3] In a recent study, levetiracetam led to enhanced LICI at ISI 50ms, but not 100ms or other tested ISIs.[3] Valproate had no effects on SICI or LICI.[3] Topiramate has been reported to enhance SICI;[4] zonisamide, with a similar mechanism of action did not affect SICI in people with idiopathic generalised epilepsy.[5] Diazepam, a $GABA_A$ receptor allosteric modulator of the benzodiazepine family, paradigmatically enhances SICI.[1] For lorazepam, no effects have been reported by some,[3] whilst others found an effect only at an ISI of 2ms but not 3ms.[6] Benzodiazepines are generally thought not to influence LICI, [1] although enhancement at ISI 50ms was recently reported.[3] To our knowledge, effects of clobazam or clonazepam on SICI or LICI have not been reported. In our sample, no difference in SICI was observed by whether benzodiazepines or topiramate were used.

References

1. Ziemann U, Reis J, Schwenkreis P, Rosanova M, Strafella A, Badawy R, et al. TMS and drugs revisited 2014. Clinical Neurophysiology 2015;126:1847–68.
2. Darmani G, Bergmann TO, Zipser C, Baur D, Müller-Dahlhaus F, Ziemann U. Effects of antiepileptic drugs on cortical excitability in humans: Effects of antiepileptic drugs on cortical excitability in humans: A TMS-EMG and TMS-EEG study. Hum Brain Mapp 2018;40:1276-1289.
3. Ruijs TQ, Heuberger JAAC, Goede AA, Ziagkos D, Otto ME, Doll RJ, et al. Transcranial magnetic stimulation as biomarker of excitability in drug development: A randomized, double-blind, placebo-controlled, cross-over study. Br J Clin Pharmacol 2022;88:2926-2937.
4. Reis J, Tergau F, Hamer HM, Müller HH, Knake S, Fritsch B, et al. Topiramate selectively decreases intracortical excitability in human motor cortex. Epilepsia 2002;43:1149–56.
5. Joo EY, Kim SH, Seo DW, Hong SB. Zonisamide decreases cortical excitability in patients with idiopathic generalized epilepsy. Clinical Neurophysiology 2008;119:1385–92.
6. Ferland MC, Therrien-Blanchet JM, Proulx S, Klees-Themens G, Bacon BA, Dang Vu TT, et al. Transcranial Magnetic Stimulation and H1-Magnetic Resonance Spectroscopy Measures of Excitation and Inhibition Following Lorazepam Administration. Neuroscience 2021;452:235–46.
